# Case Report: Spectral CT characterization of giant esophageal schwannoma with reactive lymphoid hyperplasia mimicking lymphoproliferative disease

**DOI:** 10.3389/fonc.2026.1874063

**Published:** 2026-07-03

**Authors:** Zuyi Fan, Anqi Zhang, Yumeng Xi, Meng Wang, Zhenzhen Fan, Zhihui Dong

**Affiliations:** 1 Division of Graduate, Henan Medical University, Xinxiang, China; 2 Department of Imaging, Luoyang Central Hospital, Luoyang, China; 3 Department of Pathology, Luoyang Central Hospital, Luoyang, China

**Keywords:** dual-energy CT, esophageal schwannoma, reactive lymphadenopathy, spectral CT, submucosal esophageal tumor

## Abstract

Esophageal schwannoma is an uncommon mesenchymal tumor that is frequently misdiagnosed before surgery because its clinical and radiological appearances overlap with those of other submucosal esophageal tumors and mediastinal masses. We report a 48-year-old woman with mild dysphagia and an incidentally detected right mediastinal mass. Spectral computed tomography (CT) demonstrated an 83 × 63 × 100 mm upper-to-middle thoracic esophageal submucosal mass with an intraluminal air crescent sign, heterogeneous enhancement, and multiple enlarged mediastinal and perigastric lymph nodes. Compared to conventional contrast-enhanced CT, low-energy 45-keV monoenergetic images and iodine-based spectral parameters yielded higher absolute attenuation values, objectively characterizing the heterogeneous intratumoral enhancement and demonstrating spectral differences between the primary mass and lymph nodes. Endoscopy confirmed a long submucosal elevated lesion with a localized ulcer, bronchoscopy showed extrinsic airway compression, and barium esophagography revealed a smooth filling defect. Partial esophagectomy was performed because of the tumor size and its proximity to an aberrant right subclavian artery and the brachiocephalic trunk. Due to the unachievable complete en bloc resection, the macroscopic residual mass was successfully managed with targeted adjuvant radiotherapy. Histopathology revealed a spindle-cell tumor with a prominent peripheral lymphoid cuff. To avoid diagnostic ambiguity, immunohistochemical profiling was stratified: the spindle cells were positive for S-100 and vimentin, and negative for SMA, desmin, and DOG-1, which confirmed schwannoma and excluded gastrointestinal stromal tumor. The surrounding reactive lymphoid component specifically exhibited B-cell markers.

## Introduction

Schwannoma is a benign peripheral nerve sheath tumor that may occur throughout the body, including the mediastinum, but primary esophageal schwannoma is rare. Benign primary esophageal tumors account for a small minority of esophageal tumors, and most are leiomyomas; schwannomas represent a much smaller subset (1). Systematic reviews and case-based analyses have emphasized that esophageal schwannomas most often involve the cervical, upper thoracic, or middle thoracic esophagus, occur more often in women, and are frequently difficult to diagnose before resection because endoscopic biopsy may sample only intact mucosa or nonspecific spindle-cell tissue (2, 3).

Imaging diagnosis is challenging because esophageal schwannomas can mimic leiomyoma, gastrointestinal stromal tumor, lymphoma, metastatic lymphadenopathy, and posterior mediastinal neurogenic tumors. Typical CT findings include a well-circumscribed submucosal or intramural mass that compresses rather than invades adjacent structures; however, large tumors may show heterogeneous enhancement and airway or vascular displacement (1, 4). Spectral CT, by providing low-energy monoenergetic images, iodine density, effective atomic number, and spectral attenuation curves, may add information on lesion composition and lymph node characterization. We present a giant esophageal schwannoma with marked heterogeneous enhancement and extensive reactive lymphoid hyperplasia mimicking lymphoproliferative disease, with emphasis on the incremental value of spectral CT and histopathological correlation.

## Case description

### Patient information and clinical findings

A 48-year-old woman was admitted with mild dysphagia. One month earlier, a right mediastinal mass had been incidentally detected during a periodic health examination. Past medical, personal, and family histories were negative for prior malignancy, smoking, and alcohol consumption. Systemic B symptoms, including fever, night sweats, and unexplained weight loss, were absent. Initial laboratory evaluations, encompassing a complete blood count, inflammatory parameters, and tumor markers, were within normal limits, without serological evidence of active infection or hematological malignancy. Physical examination was unremarkable, demonstrating no palpable superficial lymphadenopathy, neurological deficits, or respiratory distress.

### Timeline and spectral CT measurements

The clinical timeline and quantitative spectral CT measurements are summarized in **Table 1**.

**Table 1:** Clinical timeline and quantitative spectral CT parameters.

A. Timeline of the episode of care.		
Time point	Clinical or diagnostic event	Key finding or management decision
1 month before admission	Periodic health examination	Right mediastinal mass incidentally detected.
Admission	Clinical assessment	Mild dysphagia; physical examination otherwise unremarkable.
Initial imaging	Chest spectral CT	Large upper-to-middle thoracic esophageal submucosal mass with air crescent sign, heterogeneous enhancement, airway compression, vascular displacement, and enlarged lymph nodes.
Endoscopic and fluoroscopic assessment	Gastroscopy, bronchoscopy, and barium esophagography	Long submucosal esophageal elevation with localized ulcer; extrinsic tracheobronchial compression; smooth filling defect with rightward esophageal displacement.
Surgery	Partial esophagectomy	Large, firm, poorly mobile mass; difficult separation from aberrant right subclavian artery and brachiocephalic trunk.
Final diagnosis	Histopathology, immunohistochemistry, and Ig gene rearrangement	Esophageal schwannoma with peripheral cuff-like lymphoid infiltration and regional reactive B-cell hyperplasia; no monoclonal B-cell population detected.
Postoperative management	Surveillance	Prolonged 45-day hospitalization due to incisional fat liquefaction requiring secondary debridement.Postoperative adjuvant radiotherapy (50 Gy) administered for a residual mediastinal mass. At 5 months, normal swallowing and stable weight were observed.

B. Quantitative spectral CT parameters of the primary lesion and lymph nodes.					
ROI	Region	Conventional CT value (HU)	45-keV Monoenergetic CT value (HU)	Zeff	Iodine density (mg/mL)
S1	Highly enhancing tumor region	77	198.9	8.66	2.60
S2	Weakly enhancing tumor region	55	94.2	7.94	1.06
S3	Contralateral esophageal mucosa	35	119.7	8.30	1.78
S4	Station 2L mediastinal lymph node	53	182.2	8.56	2.38
S5	Station 7 mediastinal lymph node	50	155.4	8.29	1.80
S6	Station 2 perigastric lymph node	69	167.6	8.42	2.09

CT, computed tomography; HU, Hounsfield unit; ROI, region of interest; Zeff, effective atomic number. Station labels follow the operative/pathological records provided by the treating team.

### Diagnostic assessment

#### Spectral CT findings

Chest CT performed with a Philips Dual-layer spectral CT scanner revealed a soft-tissue mass measuring approximately 83 x 63 x 100 mm. The lesion arose from the left lateral wall of the upper-to-middle thoracic esophagus, protruded both into the esophageal lumen and mediastinum, and showed an intraluminal air crescent sign with a smooth tumor-gas interface. The non-contrast attenuation was approximately 35 HU. Contrast-enhanced CT demonstrated heterogeneous enhancement ranging from approximately 30 to 68 HU, with multiple irregular avidly enhancing foci near the esophageal submucosa measuring approximately 85 HU.

**Figure 1:**
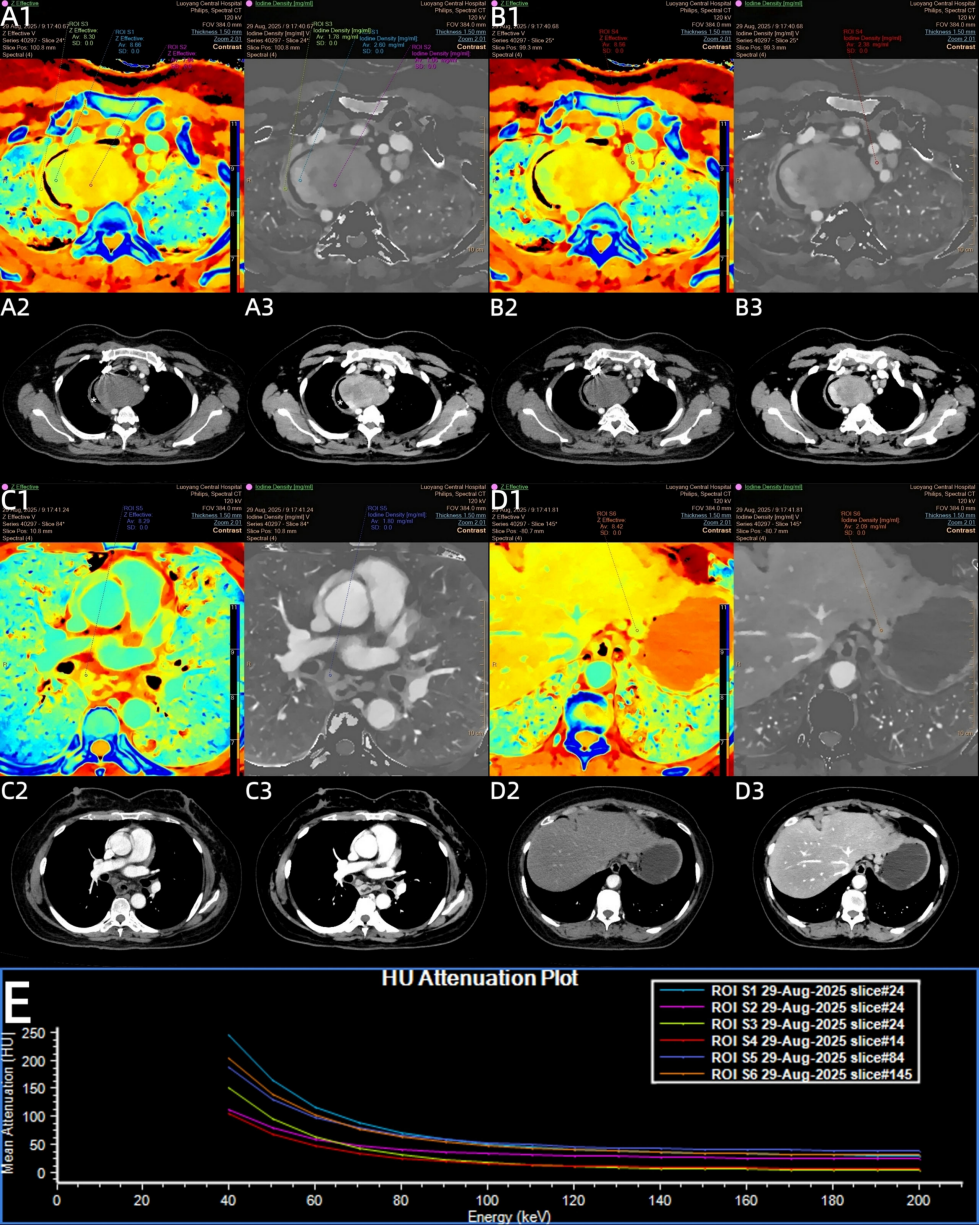
Spectral CT characterization of the primary esophageal mass and enlarged lymph nodes. **(A1-A3)** Axial spectral CT images at the same level show the primary mass between the esophagus and aortic arch on the arterial phase spectral parameter map **(A1)**, conventional image **(A2)**, and 45-keV monoenergetic image **(A3)**. The asterisks (*) in A2 and A3 explicitly indicate the air crescent sign, visualizing a smooth tumor-gas interface. Standardized regions of interest (ROIs, S1-S3) are clearly delineated. **(B1-B3, C1-C3, D1-D3)** Corresponding image types demonstrate enlarged superior mediastinal paravascular, subcarinal, and perigastric lymph nodes, respectively. **(E)** Spectral attenuation curves for ROIs S1-S6 are plotted with optimized axes for enhanced legibility; quantitative values are provided in **Table 1**.

Multiple enlarged lymph nodes were observed in the 2L, 2R, and station 7 mediastinal regions and in perigastric groups 1 and 2. The mass compressed and displaced adjacent structures, including lateral displacement of the azygos arch, deformation and narrowing of the trachea and right main bronchus, and displacement of an aberrant right subclavian artery coursing posterior to the lesion. Compared to conventional CT, the 45-keV monoenergetic images yielded substantially higher absolute attenuation values for the solid tumor components. More importantly, the relative quantitative gap between the highly and weakly enhancing intratumoral regions was significantly amplified. On conventional CT, the attenuation difference between these regions was merely 22 HU (77 HU vs. 55 HU, yielding a contrast ratio of 1.4). However, on the 45-keV monoenergetic images, this absolute difference widened substantially to 104.7 HU (198.9 HU vs. 94.2 HU, yielding a contrast ratio of 2.11). This mathematical expansion of the attenuation gap provides objective evidence that low-energy spectral CT offers a distinct advantage in characterizing profound intratumoral heterogeneity. To ensure measurement reproducibility, quantitative spectral parameters—including 45-keV monoenergetic CT attenuation, effective atomic number (Zeff), and iodine density (mg/mL)—were systematically extracted from the 45-keV monoenergetic arterial phase contrast-enhanced images and their corresponding spectral parameter maps. Concurrently, conventional CT attenuation values were measured on the corresponding conventional arterial phase contrast-enhanced images. Measurements were performed independently by two senior radiologists, each with over 10 years of experience in thoracic imaging, who were blinded to the final pathological results. Standardized circular regions of interest (ROIs, ranging from 10 to 20 mm²) were manually delineated on the solid portions of the primary tumor (highly and weakly enhancing regions), the contralateral normal esophageal mucosa, and the enlarged lymph nodes. Care was taken to strictly avoid adjacent vascular structures, intraluminal gas, calcifications, and macroscopic necrotic or cystic areas. For each target structure, measurements were repeated three times across consecutive axial slices, and the values obtained by both radiologists were ultimately averaged to yield the final quantitative data. Analysis of these extracted data revealed distinct quantitative patterns among the measured regions. Specifically, the highly enhancing tumor region exhibited the highest spectral values, with an iodine density (2.60 mg/mL) approximately 2.45 times greater than that of the weakly enhancing region (1.06 mg/mL). The enlarged lymph nodes demonstrated intermediate iodine densities (ranging from 1.80 to 2.38 mg/mL), which were directionally lower than the highly enhancing tumor region but distinctly higher than both the weakly enhancing region and the normal contralateral esophageal mucosa (1.78 mg/mL) (**Table 1**; **Figure 1**).

#### Endoscopic, bronchoscopic, and fluoroscopic findings

**Figure 2:**
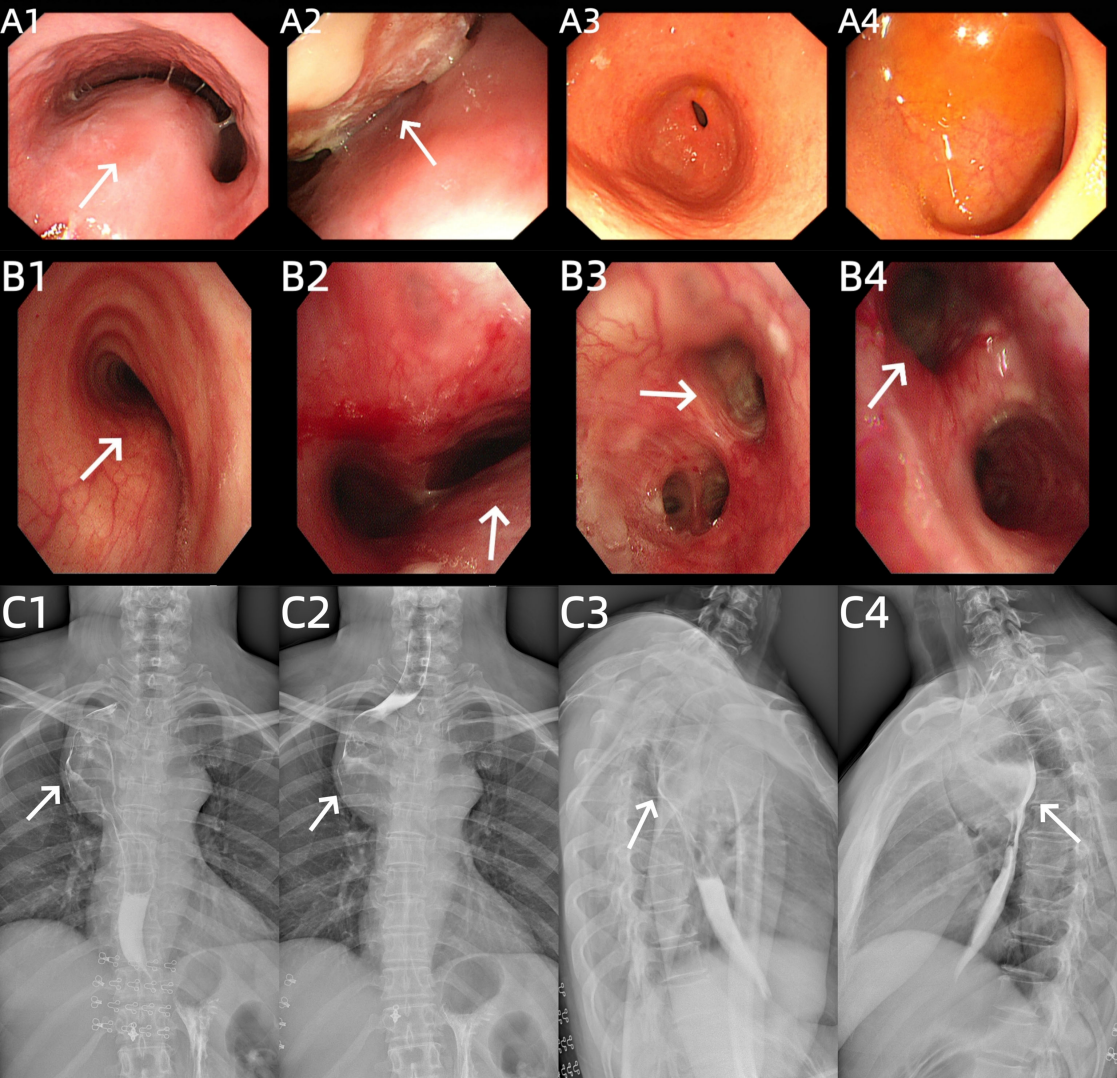
Endoscopic, bronchoscopic, and barium esophagographic findings. **(A1-A4)** Upper gastrointestinal endoscopy demonstrates a patent esophageal lumen with a long submucosal elevated lesion located 20–30 cm from the incisors (arrow in **A1**) and a localized mucosal ulceration (arrow in **A2**). The antrum **(A3)** and duodenum **(A4)** are unremarkable. **(B1-B4)** Bronchoscopy reveals severe extrinsic airway compression; distinct arrows highlight the high-grade narrowing of the middle-to-distal trachea **(B1)** and the profound distortion and stenosis of the right main bronchus orifice **(B2-B4)**. **(C1-C4)** Barium esophagography across multiple projections illustrates the massive mass effect. Anteroposterior views demonstrate marked lateral displacement of the esophagus (arrows in **C1, C2**), while lateral/oblique views clearly delineate a massive, smooth esophageal filling defect indicative of extrinsic compression (arrows in **C3, C4**).

Gastroscopy demonstrated a patent esophageal lumen with an elevated, approximately 10-cm-long submucosal mass located 20–30 cm from the incisors. The mucosal surface was smooth overall, with a localized ulcer. Histopathological evaluation of five endoscopic mucosal biopsy specimens demonstrated chronic inflammation without evidence of neoplasia. Barium esophagography showed an upper-to-middle esophageal filling defect, smooth wall contours, irregular mucosal folds, and rightward displacement of the esophagus. Bronchoscopy revealed severe narrowing of the middle-to-distal trachea, distortion of the carina, and stenosis of the right main bronchial orifice due to extrinsic compression (**Figure 2**). Advanced tissue sampling techniques, including endoscopic ultrasound-guided fine-needle aspiration (EUS-FNA) and regional lymph node biopsy, were omitted based on specific clinical and anatomical criteria. The high-grade extrinsic tracheobronchial compression established an immediate indication for surgical decompressing intervention. Furthermore, computed tomography delineated a close spatial relationship between the mass and major vascular structures, including the brachiocephalic trunk and an aberrant right subclavian artery, which presented an elevated risk of hemorrhagic complications for blind or deep needle interventions. Given that surgical resection was independently mandated by both the critical mechanical airway obstruction and the absolute mass volume, proceeding directly to operative intervention without additional preoperative tissue sampling was considered the optimal clinical pathway.

#### Pathological and molecular assessment

Intraoperative frozen-section examination showed proliferating muscle fibers with edema, patchy lymphocytic infiltration, and focal disordered fibrous hyperplasia resembling a tumor-like proliferation. Final pathological evaluation of the resected specimen confirmed a spindle-cell tumor with tumor-free esophageal and gastric luminal surgical margins. To avoid diagnostic ambiguity, the immunohistochemical profile was strictly stratified between the primary spindle cells and the surrounding microenvironment. The spindle tumor cells demonstrated diffuse positivity for S-100 and vimentin, while being definitively negative for smooth muscle actin (SMA), desmin, and DOG-1, with CD34 solely highlighting the adjacent vascular endothelium. This profile established the diagnosis of esophageal schwannoma and excluded leiomyoma and gastrointestinal stromal tumor. Crucially, despite a focally elevated Ki-67 proliferation index of approximately 10%, rigorous histological examination revealed an absence of severe cellular atypia, coagulative necrosis, and elevated mitotic activity, thereby excluding the possibility of a malignant peripheral nerve sheath tumor (MPNST). Furthermore, the prominent cuff-like lymphocytic infiltrate at the tumor periphery was independently characterized. This reactive lymphoid component—distinct from the spindle cells—exhibited positivity for B-cell markers (CD20, PAX-5) and Bcl-2, with preserved follicular dendritic cell networks (CD21+, CD23+).

**Figure 3:**
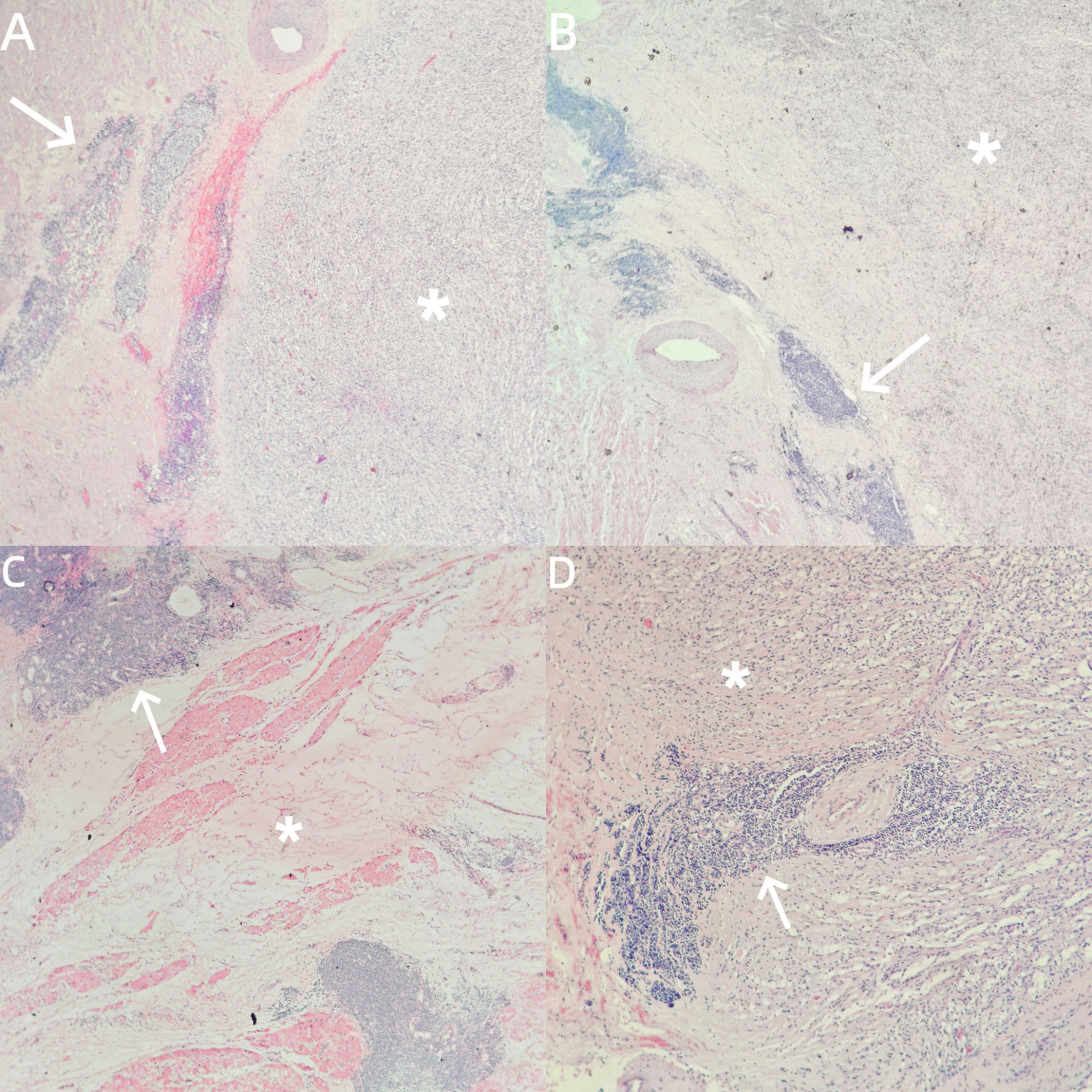
Histopathological features of esophageal schwannoma with peripheral lymphoid cuffing. **(A-D)** Hematoxylin and eosin (H&E) stained sections (original magnification, ×40). The central tumor component, consisting of haphazardly arranged spindle cells, is indicated by asterisks (*). The tumor periphery is surrounded by a prominent cuff-like lymphoid infiltrate, which is explicitly highlighted by arrows, demonstrating the clear histological boundary between the primary schwannoma and the reactive B-cell lymphoid hyperplasia.

Regional lymphoid hyperplasia involving the mediastinal and perigastric lymph nodes initially raised concern for marginal zone lymphoma. However, immunoglobulin gene rearrangement analysis demonstrated no monoclonal B-cell population, supporting reactive hyperplasia rather than lymphoma (**Figure 3**).

#### Therapeutic intervention

A cervical-thoracic-abdominal three-incision approach was utilized to perform the partial esophagectomy and concurrent adhesiolysis. Intraoperative exploration identified a firm, poorly mobile upper thoracic esophageal mass measuring approximately 10 cm in length. The superior aspect of the tumor was intimately adherent to the aberrant right subclavian artery and brachiocephalic trunk, rendering the dissection technically challenging. The decision to perform a partial esophagectomy rather than a simple enucleation was strictly driven by the massive tumor size, broad muscular involvement, proximity to major vessels, and critical airway compression. Following resection, digestive tract reconstruction was achieved via an esophagogastric cervical anastomosis. Concurrently, regional lymph node sampling was performed, yielding 21 perigastric lymph nodes. Final pathological assessment confirmed tumor-free esophageal and gastric luminal surgical margins. However, due to the tumor’s intimate adherence to the aberrant right subclavian artery and brachiocephalic trunk, a complete en bloc R0 resection was technically unachievable, unavoidably leaving a macroscopic residual mass at the vascular border. Postoperatively, the patient was initially managed in the intensive care unit before transitioning to the general ward. The early recovery phase was complicated by incisional fat liquefaction, which was successfully managed with secondary debridement and suturing.

### Follow-up and outcomes

Given the large tumor size and a Ki-67 proliferation index of approximately 10%, a rigorous surveillance strategy was implemented. The initial postoperative course was complicated by incisional fat liquefaction requiring secondary debridement and suturing, extending the hospital stay to 45 days. Follow-up computed tomography postoperatively confirmed a prominent residual soft-tissue mass (measuring approximately 53 × 43 mm) located below the anastomosis. To achieve local control of this residual disease, the patient was administered targeted adjuvant radiotherapy (50 Gy in 25 fractions). At the 5-month follow-up, the patient demonstrated complete resolution of dysphagia and maintained a stable nutritional status (body mass index: 23.4 kg/m²). Subsequent computed tomography confirmed stable local postoperative changes without tumor progression and the regression of previously enlarged regional lymph nodes. Nevertheless, the relatively short follow-up duration (5 months) remains a limitation in comprehensively evaluating the long-term recurrence risk.

### Patient perspective

“Before the surgery, I was highly anxious after a routine health examination unexpectedly revealed a giant chest tumor, which explained my progressively worsening swallowing difficulties and shortness of breath. The surgical recovery was intensely challenging, particularly due to the prolonged 45-day hospitalization and unexpected wound healing complications. However, the multidisciplinary team provided meticulous care. Now, five months after the operation and subsequent adjuvant radiotherapy, my swallowing function has completely returned to normal, and my body weight remains stable. Although the postoperative journey was arduous, I am profoundly grateful for the treatment that successfully relieved my severe symptoms and restored my daily quality of life”.

## Discussion

This case is notable for three reasons. First, the tumor was giant and located in the upper-to-middle thoracic esophagus, producing airway compression and complex vascular relationships. Second, the lesion showed heterogeneous enhancement, with the highly enhancing regions demonstrating a steep upward trajectory on the spectral attenuation curve at lower energy levels (e.g., 45 keV) compared to weakly enhancing regions. Third, multiple enlarged lymph nodes and peripheral B-cell-rich lymphoid cuffing mimicked lymphoproliferative disease, yet molecular testing supported reactive hyperplasia. These features created a challenging diagnostic scenario relevant to radiologists, oncologists, surgeons, gastroenterologists, and pathologists.

Esophageal schwannomas are rare and are often diagnosed only after surgical resection. In a systematic review from mainland China, most lesions arose in the neck, upper esophagus, or middle thoracic esophagus; the authors emphasized high rates of clinical misdiagnosis and the need for immunohistochemistry, especially S-100 positivity, for definitive diagnosis (2). Another case-based review similarly underscored that immunohistochemistry is essential, with S-100 positivity and lack of smooth muscle or gastrointestinal stromal tumor markers supporting schwannoma (3). Our case aligns with this literature in terms of location, spindle-cell morphology, and S-100 positivity, but adds spectral CT-pathology correlation and a nodal mimic of lymphoma.

The main radiologic differential diagnosis is leiomyoma, the most common benign submucosal tumor of the esophagus. Leiomyomas more often arise in the mid-to-distal esophagus and typically show mild homogeneous enhancement. In contrast, schwannomas are frequently reported in the upper or middle esophagus and may be associated with lymphoid cuffing. Gastrointestinal stromal tumor is another differential diagnosis, particularly for a large enhancing spindle-cell tumor, but the present tumor was DOG-1 negative and showed S-100 positivity. Mediastinal neurogenic tumor, metastatic nodal disease, and lymphoma should also be considered when a large mediastinal mass and nodal enlargement are present.

The air crescent sign in this case reflected protrusion of the submucosal tumor into the esophageal lumen, creating a smooth interface with intraluminal gas. The absence of overt infiltration and the tendency to compress and displace adjacent structures were consistent with benign behavior. Nevertheless, tumor size, airway narrowing, and intimate relationships with vascular structures increased the surgical complexity. Previous reports have indicated that enucleation may be adequate for small benign lesions, whereas larger tumors with broad muscular involvement may require esophagectomy or segmental resection because of the risk of mucosal defect, stricture, or leakage after enucleation (1, 5).

A distinctive feature of this case was the quantitative spectral CT characterization, which provided significant added value over conventional contrast-enhanced CT. While conventional CT merely visually identifies mixed attenuation, spectral CT objectively quantifies local tissue vascularity and composition to refine the differential diagnosis. Existing literature indicates that typical esophageal leiomyomas exhibit globally low normalized iodine concentrations (NIC) (e.g., a reported median NIC of 1.120 in the portal phase) and relatively flat spectral attenuation curves due to their hypovascular smooth muscle composition (7). Conversely, gastrointestinal stromal tumors (GISTs) are distinctly hypervascular overall, often demonstrating significantly higher global NIC values (e.g., a reported median NIC of 2.726) and steep spectral trajectories (7). Furthermore, high-risk GISTs consistently display elevated global NIC values that correlate with increased tumor angiogenesis and permeability (8). Esophageal or mediastinal lymphomas generally present as uniformly hypercellular masses with consistent, moderate global iodine concentrations.

In this case, our giant primary schwannoma presented a profound macroscopic dual-pattern, characterized by a massive internal quantitative variance (the absolute iodine density of the highly enhancing region was approximately 2.45 times greater than that of the weakly enhancing region). However, a significant methodological limitation exists in utilizing this variance for definitive differential diagnosis. Existing literature predominantly characterizes typical leiomyomas, GISTs, and lymphomas using global quantitative parameters (e.g., reported global median NIC values for leiomyomas and GISTs). Directly comparing these global averages against the internal regional variance of our case constitutes an asymmetric comparison. Furthermore, as large, high-risk GISTs frequently undergo necrosis or hemorrhage, they can also demonstrate significant intratumoral heterogeneity. Because current literature lacks explicitly reported regional quantitative ratios (e.g., iodine density differences between hypervascular and hypovascular regions) for these mesenchymal mimics, an ‘apples-to-apples’ comparative analysis cannot be robustly established. Consequently, the profound internal quantitative disparity observed in our case should be explicitly viewed as hypothesis-generating rather than conclusive. It underscores the capability of spectral CT to objectively map intratumoral microvascular heterogeneity, but whether this specific highly disparate spectral signature serves as a specific biomarker for degenerating schwannomas requires future validation through large-cohort comparative studies.

In this case, the enlarged lymph nodes exhibited intermediate quantitative spectral parameters (e.g., iodine densities of 1.80–2.38 mg/mL) that overlapped with the primary tumor’s highly heterogeneous values, rather than presenting a distinctly separate spectral signature. Because spectral CT parameters may overlap between reactive and neoplastic processes, imaging alone is insufficient to reliably establish nodal benignity. Consequently, definitive diagnosis strictly relied on direct histopathological and molecular confirmation. Histopathological examination of the 21 resected regional lymph nodes explicitly confirmed reactive chronic inflammation. Furthermore, immunoglobulin gene rearrangement analysis of the lymphoid tissue demonstrated a definitive polyclonal pattern with no monoclonal B-cell population (**Supplementary File 1**), providing absolute molecular confirmation of reactive lymphoid hyperplasia rather than lymphoproliferative disease.

The pathological finding of peripheral cuff-like lymphoid infiltration is important. In gastrointestinal schwannomas, lymphoid cuffing is a recognized feature and can be prominent enough to raise concern for lymphoma or nodal metastasis, particularly when regional lymphadenopathy is present. In the present case, the lymphoid component showed B-cell lineage, but absence of monoclonal immunoglobulin gene rearrangement supported a reactive process. Recognizing this possibility may help prevent overstaging and guide appropriate pathological work-up.

The clinical course of most esophageal schwannomas is favorable after complete resection. Malignant transformation or malignant peripheral nerve sheath tumor of the esophagus is rare but has been reported (6). Therefore, careful pathological assessment of cellular atypia, mitotic activity, necrosis, tumor margins, and Ki-67 index is necessary. Given the presence of a macroscopic residual mediastinal mass and a focally elevated Ki-67 index of approximately 10%, the administration of postoperative adjuvant radiotherapy followed by rigorous surveillance was implemented as a rational multidisciplinary strategy, even though definitive malignant features were absent.

This report has limitations. It describes a single case, so the diagnostic value of spectral CT cannot be generalized. Preoperative tissue diagnosis was not definitive, and the precise pathological correlate of each spectral CT region cannot be proven without region-by-region whole-mount mapping. Despite these limitations, the case demonstrates a practical imaging-pathology framework for rare esophageal schwannoma complicated by reactive lymphadenopathy.

## Conclusion

In conclusion, this case underscores the pivotal clinical added value of quantitative spectral CT in the pre-operative management of rare, giant esophageal mesenchymal tumors. Beyond conventional morphological delineation, spectral CT significantly enhances preoperative diagnostic confidence by mathematically amplifying and quantifying subtle intratumoral heterogeneities. Specifically, identifying a distinct macroscopic dual-pattern—characterized by a marked expansion of the 45-keV attenuation gap (104.7 HU) and a 2.45-fold disparity in iodine density—provides a precise quantitative map of profound internal heterogeneity. Although its definitive role as a differential imaging biomarker remains hypothesis-generating and necessitates further validation against other heterogeneous mimics, these quantitative findings successfully translated into tangible refinements in treatment planning.

These quantitative findings successfully translated into tangible refinements in treatment planning. By accurately mapping the highly disparate internal microvascular architecture of this massive 10-cm lesion in immediate proximity to the aberrant right subclavian artery and brachiocephalic trunk, the spectral parameters provided the multidisciplinary team with a rigorous risk-assessment framework. These objective data justified the clinical decision to eschew a high-risk simple tumor enucleation in favor of a safer, planned partial esophagectomy, and subsequently guided the precise implementation of targeted adjuvant radiotherapy to address microscopic residual margins. Consequently, spectral CT effectively bridges the gap between quantitative imaging science and precision surgical ontology in complex mediastinal interventions.

## Data Availability

The original contributions presented in the study are included in the article/**Supplementary Material**. Further inquiries can be directed to the corresponding author.
