# Supplementary material for: Case Report: Spectral CT characterization of giant esophageal schwannoma with reactive lymphoid hyperplasia mimicking lymphoproliferative disease
Source: Front Oncol. 2026 Jul 3;16:1874063. doi: 10.3389/fonc.2026.1874063 (PMC13375467; doi:10.3389/fonc.2026.1874063)

**Supplementary Figure 1: Immunoglobulin (Ig) Gene Rearrangement Analysis**

**Report Type:** Lymphoma Ig Gene Rearrangement Test Report

**Figure Legend:** The multiplex fluorescent polymerase chain reaction (PCR) combined with capillary electrophoresis was utilized to analyze the lymphoid tissue. Panels show the detection profiles across the IGH, IGK, and IGL loci. All analyzed targets demonstrate a definitive polyclonal pattern with overlapping, multi-peak curves, convincingly ruling out a monoclonal B-cell lymphoproliferative disease. ***Note: The original laboratory report is in Chinese; patient-identifying descriptors and institutional tracking numbers have been permanently redacted and cropped out to strictly guarantee patient privacy in accordance with international ethical standards.***

**Key Laboratory Findings (Translated):**

**Institution:** The First Affiliated Hospital of Zhengzhou University

**Target Cells:** ≥200 cells

**Sample:** Esophageal mass (reactive lymphoid hyperplasia)

**Conclusion:** No prominent monoclonal B-cell population proliferation was detected. Findings support a reactive process.

**IGH, IGL, IGK Loci:** Polyclonal Rearrangement


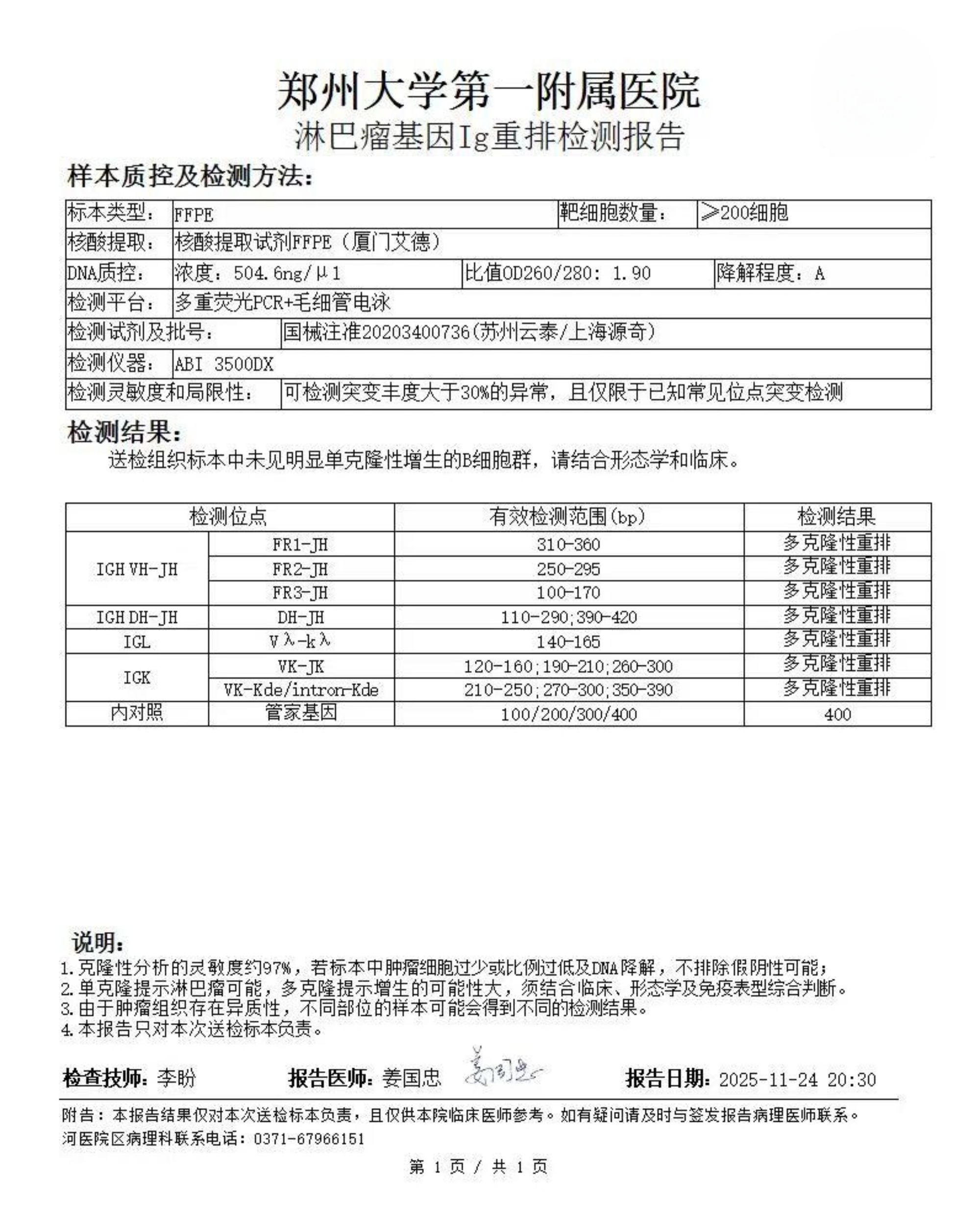

Supplement: Supplementary file 1 [file DataSheet1.doc]
